# Phenotyping diabetic cardiomyopathy in Europeans and South Asians

**DOI:** 10.1186/s12933-019-0940-z

**Published:** 2019-10-11

**Authors:** Elisabeth H. M. Paiman, Huub J. van Eyk, Maurice B. Bizino, Ilona A. Dekkers, Paul de Heer, Johannes W. A. Smit, Ingrid M. Jazet, Hildo J. Lamb

**Affiliations:** 1 Dept. Radiology, Leiden University Medical Center, P.O. Box 9600, Postal Zone C2-S, 2300 RC Leiden, The Netherlands; 2 Dept. Internal Medicine, Leiden University Medical Center, P.O. Box 9600, Postal Zone C7-Q, 2300 RC Leiden, The Netherlands; 3 Dept. Internal Medicine, Radboud University Medical Center, P.O. Box 9101, 6500 HB Nijmegen, The Netherlands

**Keywords:** Diabetes mellitus, type 2, Diabetic cardiomyopathies, Myocardial steatosis, Myocardial diffuse fibrosis, Proton-magnetic resonance spectroscopy, T1 mapping, European, South Asian

## Abstract

**Background:**

The pathogenesis and cardiovascular impact of type 2 diabetes (T2D) may be different in South Asians compared with other ethnic groups. The phenotypic characterization of diabetic cardiomyopathy remains debated and little is known regarding differences in T2D-related cardiovascular remodeling across ethnicities. We aimed to characterize the differences in left ventricular (LV) diastolic and systolic function, LV structure, myocardial tissue characteristics and aortic stiffness between T2D patients and controls and to assess the differences in T2D-related cardiovascular remodeling between South Asians and Europeans.

**Methods:**

T2D patients and controls of South Asian and European descent underwent 3 Tesla cardiovascular magnetic resonance imaging (CMR) and cardiac proton-magnetic resonance spectroscopy (^1^H-MRS). Differences in cardiovascular parameters between T2D patients and controls were examined using ANCOVA and were reported as mean (95% CI). Ethnic group comparisons in the association of T2D with cardiovascular remodeling were made by adding the interaction term between ethnicity and diabetes status to the model.

**Results:**

A total of 131 individuals were included (54 South Asians [50.1 ± 8.7 years, 33% men, 33 patients vs. 21 controls) and 77 Europeans (58.8 ± 7.0 years, 56% men, 48 patients vs. 29 controls)]. The ratio of the transmitral early and late peak filling rate (E/A) was lower in T2D patients compared with controls, in South Asians [− 0.20 (− 0.36; − 0.03), *P *= 0.021] and Europeans [− 0.20 (− 0.36; − 0.04), *P *= 0.017], whereas global longitudinal strain and aortic pulse wave velocity were similar. South Asian T2D patients had a higher LV mass [+ 22 g (15; 30), *P *< 0.001] (*P* for interaction by ethnicity = 0.005) with a lower extracellular volume fraction [− 1.9% (− 3.4; − 0.4), *P *= 0.013] (*P* for interaction = 0.114), whilst European T2D patients had a higher myocardial triglyceride content [+ 0.59% (0.35; 0.84), *P *= 0.001] (*P* for interaction = 0.002) than their control group.

**Conclusions:**

Diabetic cardiomyopathy was characterized by impaired LV diastolic function in South Asians and Europeans. Increased LV mass was solely observed among South Asian T2D patients, whereas differences in myocardial triglyceride content between T2D patients and controls were only present in the European cohort. The diabetic cardiomyopathy phenotype may differ between subsets of T2D patients, for example across ethnicities, and tailored strategies for T2D management may be required.

## Background

Type 2 diabetes (T2D), independent of other cardiovascular risk factors, is associated with an increased risk of heart failure [1]. South Asians, who represent 20% of the world’s population, are at particular risk of developing T2D [2, 3]. Individuals of South Asian descent appear to have a metabolically disadvantageous phenotype with a relatively high total body fat percentage [4, 5]. Also, the metabolic sensitivity to excess fat mass may be more pronounced among South Asians compared with other ethnic groups, as indicated by increased insulin resistance at similar adiposity levels [6, 7]. In individuals of South Asian descent, the release of adipose tissue metabolites may be disturbed [8]. Also, in South Asians in particular, excess fat mass may cause a state of chronic inflammation [9]. Importantly, the impact of T2D on cardiac function may be greater among South Asians compared with Europeans [10]. However, most previous studies on diabetic cardiomyopathy were performed in white populations or ethnicity was not reported [11–13]. Little is known regarding the differences in the diabetic cardiomyopathy phenotype across ethnicities, whereas increased insight into the ethnic-specific cardiovascular consequences may guide the development of tailored strategies for the management of T2D.

By using cardiovascular magnetic resonance (CMR), the impact of T2D on the left ventricle (LV) can be characterized on a functional, structural and myocardial tissue level. Strain echocardiography studies have shown that not merely LV diastolic function, which may be most susceptible to myocardial energy depletion, but also LV systolic function might be impaired [14]. The introduction of feature tracking has enabled the assessment of both longitudinal and circumferential strain based on standard cine images, which can be considered more sensitive measures of myocardial contractility compared with LV ejection fraction [15]. Myocardial diffuse fibrosis, as the result of hyperglycemia and systemic inflammation in T2D, may be an early hallmark of LV remodeling, preceding functional impairments [16]. Also, myocardial steatosis has been proposed as an important contributing factor to both structural and functional cardiac remodeling in patients with T2D [12–14]. In this study, we aimed to characterize the differences in LV diastolic and systolic function, LV structure and myocardial tissue characteristics and aortic stiffness between T2D patients and controls and to assess the differences in T2D-related cardiovascular remodeling between South Asians and Europeans.

## Methods

### Study population

This is a single-center, cross-sectional study. The data of the T2D patients of European and South Asian origin (i.e. Hindustani Surinamese, Indian, Pakistani, Bangladeshi or Sri Lankan) were derived from the baseline measurements of two previous randomized controlled trials (ClinicalTrials.gov NCT01761318 [17] and NCT02660047 [18], respectively). In addition, healthy controls of European and South Asian descent in the same age range and with a similar sex distribution as compared with the T2D patients were prospectively enrolled. Ethnicity was based on self-identified origin and self-reported origin of both biological parents and their ancestors. Written informed consent was obtained prior to inclusion. The study complied with the revised Declaration of Helsinki and was approved by the institutional review board (Leiden University Medical Center, Leiden, The Netherlands).

All T2D patients were obese [body mass index (BMI) ≥ 25 kg/m^2^ for Europeans and ≥ 23 kg/m^2^ for South Asians] and used metformin, sulfonylurea derivatives and/or insulin. Initially, the inclusion criteria for the European and South Asian study population were similar. However, due to the insufficient number of eligible T2D patients, the inclusion criteria for the South Asians were adjusted. For the European and South Asian T2D patients, respectively, age ranged from 18–70 and 18–75 year, glycated hemoglobin (HbA1c) was ≥ 52.5 and < 86.5 mmol/mol (≥ 7.0 and ≤ 10.0%) and ≥ 47.5 and < 96.5 mmol/mol (≥ 6.5 and ≤ 11.0%), systolic and diastolic blood pressure was < 150/85 mmHg and < 180/110 mmHg, estimated glomerular filtration rate (eGFR) was > 60 mL/min/1.73 m^2^ and > 30 mL/min/1.73 m^2^, no history of significant coronary artery disease for the European T2D group (significant coronary artery disease was defined as: a history of coronary artery bypass grafting and/or percutaneous coronary intervention or significant coronary artery stenosis proven by coronary angiography or non-invasive imaging), and no acute coronary accident in the preceding 30 days for the South Asian T2D group. Main exclusion criteria were: any contra-indication for contrast-enhanced CMR and heart failure New York Heart Association class III–IV. All T2D patients were screened for abnormalities on rest echocardiography (ECG). For the present study, all T2D patients with significant coronary artery disease or valvular disease were excluded.

The healthy controls were recruited by advertisements in Leiden University Medical Center (Leiden, The Netherlands) and in local newspapers. Individuals aged 40–70 years without a history of cardiovascular disease, no medication use and no contra-indications for contrast-enhanced CMR were eligible for participation in the healthy control group. Exclusion criteria were: prediabetes or diabetes [fasting glucose ≥ 6.1 mmol/L, 2-h glucose after 75 g glucose ≥ 7.8 mmol/L or HbA1c ≥ 39 mmol/mol (≥ 5.7%)], metabolic syndrome {≥ 2 of the following criteria: systolic and diastolic blood pressure blood pressure ≥ 140/90 mmHg; triglycerides ≥ 1.7 mmol/L; HDL-cholesterol < 0.9 mmol/L for men or < 1.0 mmol/L for women; obesity (BMI ≥ 30 kg/m^2^) or abdominal obesity [waist/hip ratio: > 0.9 for men or > 0.85 for women or waist circumference: ≥ 102 cm for men or ≥ 88 cm for women (Europeans); ≥ 90 cm for men or ≥ 80 cm for women (South Asians)]}, abnormalities upon physical examination, laboratory assessment (blood count, liver and kidney function) or rest ECG.

### Data collection

Study participants were included after a screening visit. Clinical and CMR examinations were scheduled either in the morning or evening, after an overnight or 6 h fast, respectively (for T2D patients, the insulin dose was adjusted and other glucose-lowering medication was temporarily discontinued). Smoking status was self-reported and was categorized as currently vs. previously or never. Blood pressure was measured in seated position on the right arm after rest, using a validated automatic oscillometric device (SureSigns VS3, Philips, Best, The Netherlands) and was the mean of two consecutive measurements. HbA1c was examined with ion-exchange high-performance liquid chromatography (HPLC; Tosoh G8, Sysmex Nederland B.V., Etten-Leur, The Netherlands). Lipid levels were assessed on a Modular P800 analyzer (Roche Diagnostics, Mannheim, Germany) with calculation of low-density lipoprotein (LDL)-cholesterol according to the Friedewald formula. The total body fat percentage was derived from bioelectrical impedance analysis (BIA; Bodystat 1500, Bodystart Ltd., Douglas, United Kingdom).

### CMR acquisition and analysis

CMR scans were acquired using a 3 Tesla MR scanner with a dStream Torso anterior coil and a FlexCoverage posterior coil, with up to 32 coil elements for signal reception (Ingenia, Philips, Best, The Netherlands).

### VAT and abdominal SAT

Visceral and abdominal subcutaneous adipose tissue (VAT and abdominal SAT) were assessed on 2-point Dixon water-fat separated transverse images of the abdomen. VAT and abdominal SAT were semi-automatically measured based on pixel intensity thresholding on three reformatted transverse slices at the level of the fourth and fifth lumbar vertebrae, with slice thickness of 10 mm and slice gap of 12 mm (repetition time (TR) 3.5 ms, first/second echo time (TE) 1.19/2.3 ms, flip angle (FA) 10°, field of view (FOV) 500 × 365 mm^2^, acquired voxel size 1.60 × 1.70 mm^2^, slice thickness 4 mm, slice gap − 2 mm, number of slices 140). VAT and abdominal SAT were quantified as the mean area in squared centimeters of all three slices (MASS version 2015-EXP, Leiden University Medical Center, Leiden, The Netherlands).

### LV systolic and diastolic function

LV function was examined on breath-hold ECG-triggered short-axis and 2-, 3- and 4-chamber long-axis cine balanced steady-state free precession [TE/TR 1.5/3.0 ms, FA 45°, FOV 350 × 350 mm^2^ (4-chamber) and 400 × 352 mm^2^ (short-axis), acquired voxel size 2.0 × 1.6 mm^2^ (4-chamber) and 1.5 × 1.5 mm^2^ (short-axis), slice thickness 8 mm, number of phases 30 (4-chamber) and 35 (short-axis)] and free-breathing ECG-gated whole-heart gradient-echo 4D velocity-encoded MR [venc 150 cm/s, TE/TR 4.6/9.0 ms, FA 10°, FOV 360 × 360 mm^2^, acquired voxel size 3.0 × 3.0 mm^2^, slice thickness 3 mm, number of slices 41, number of phases 30, sensitivity encoding (SENSE) factor 2].

LV systolic function parameters included LV ejection fraction measured on short-axis cines (MASS version 2015-EXP, Leiden University Medical Center, Leiden, The Netherlands) and global longitudinal and circumferential strain (GLS and GCS) derived from long-axis and short-axis cines using feature tracking (QStrain 2.0, Medis Suite 3.0, Medis medical imaging systems, Leiden, The Netherlands). LV contours were semi-automatically drawn in the short-axis images in the end-diastolic and end-systolic phase, to quantify the end-diastolic LV mass, LV end-diastolic and end-systolic volumes, LV stroke volume, LV ejection fraction, cardiac output and cardiac index. GLS was the average of the peak systolic strain on 2-, 3- and 4-chamber cines. GCS was the peak systolic strain in the mid-ventricular short-axis cines.

LV diastolic strain parameters included the global longitudinal early peak diastolic strain rate (GLSR-E) (average of the early peak diastolic strain rate in 2-, 3- and 4-chamber view) and the global circumferential early peak diastolic strain rate (GCSR-E) (the early peak diastolic strain rate in the mid-ventricular short-axis cines). LV diastolic function parameters derived from 4D velocity-encoded MR included the ratio of the transmitral early (E) and late (A) peak filling rate (E/A ratio), the peak deceleration slope of the E wave (E dec peak), the estimated LV filling pressure (the ratio of the transmitral early peak velocity and the early peak diastolic mitral septal tissue velocity (Ea) measured on 4-chamber cines) and the estimated LV compliance (the ratio of LV end-diastolic volume and the estimated LV filling pressure) [19]. The transmitral flow rate curves were constructed after retrospective mitral valve tracking, perpendicular to the streamlines of inflow across the mitral valve, at the location of peak flow velocity, with correction for through-plane motion of the LV myocardial wall (MASS version 2015-EXP, Leiden University Medical Center, Leiden, The Netherlands) [20, 21].

### Aortic stiffness

Aortic stiffness was quantified by the aortic pulse wave velocity, which was derived from a double-oblique aorta scout view and two free-breathing 2D velocity-encoded MR scans at the level of the ascending and abdominal aorta (venc 200 cm/s and 150 cm/s, respectively; TE/TR 2.5/4.4 ms, FA 20°, FOV 350 × 282 mm^2^, slice thickness 8 mm, acquired voxel size 2.8 × 2.8 mm^2^, temporal resolution 10 ms). The aortic pulse wave velocity was calculated by dividing the distance between ascending and abdominal aorta by the transit time of the systolic wave front (MASS version 2015-EXP, Leiden University Medical Center, Leiden, The Netherlands and in-house developed software) [22].

### Myocardial steatosis

Myocardial steatosis was examined using ECG-triggered, respiratory-navigated cardiac proton-magnetic resonance spectroscopy (^1^H-MRS) in a voxel of 40 × 15 × 25 mm^3^ in the mid-ventricular septum, using a high permittivity pad on the thorax [TE 35 ms, TR 3.5 or 9 s (water-suppressed and non-water suppressed acquisition, respectively), acquired samples 2048 (spectral resolution 0.73 Hz/sample), number of signal averages 64 or 6 (water-suppressed and non-water suppressed acquisition, respectively)] [23]. Starting values for the fit of the acquired spectrum were: triglyceride methyl (CH_3_) 0.9 ppm, triglyceride methylene (CH_2_)^n^ 1.3 ppm, COO–CH_2_ 2.05 ppm, creatine 3.05 ppm, trimethylamines (TMA) 3.25 ppm. The myocardial triglyceride content was quantified as the amplitude of (CH_2_)^n^ divided by the amplitude of unsuppressed water, multiplied by 100% [24] (in-house developed software and the Java-based MR user interface, jMRUI v5.0; MRUI Consortium) [25, 26].

### Myocardial diffuse fibrosis

Myocardial diffuse fibrosis was quantified using native and post-contrast modified Look-Locker inversion recovery (MOLLI) T1 mapping, obtained in short-axis orientation at the mid-ventricular level (TE/TR 1.1/2.3 ms, FA 20°, FOV 350 × 300 mm^2^, slice thickness 8 mm, acquired voxel size 2.1 × 2.1 mm^2^, SENSE factor 2). Post-contrast T1 mapping was acquired 20–25 min after administration of 0.15 mmol gadoterate meglumine (0.5 mmol/mL Dotarem; Guerbet Villepinte, France) per kilogram of body weight. Because of ongoing optimization of the T1 mapping protocols throughout the study, native and post-contrast MOLLI acquisition schemes were adjusted (for the European T2D patients: 3b(3b)3b(3b)5b and 3b(3b)3b(3b)5b; for the European controls: 3b(3s)3b(3s)5b and 3b(3s)3b(3s)5b; for the South Asian T2D patients and controls: 5s(3s)3s and 4s(1s)3s(1s)2s, respectively). T1 relaxation times were measured in the mid-ventricular septum, after manual correction for motion (QMap 2.2.18, Medis Suite 3.0, Medis medical imaging systems, Leiden, The Netherlands).

### Statistical analysis

Clinical characteristics, adiposity and cardiovascular parameters were presented for the T2D and control group, for Europeans and South Asians separately, and were expressed as mean ± SD, medians (interquartile ranges) or numbers (percentages). We assessed the differences in clinical characteristics and cardiovascular parameters between T2D patients and controls, for Europeans and South Asians, using the Student’s t-test or the Fisher’s exact test, and reported the differences in adiposity parameters as means (95% CI). Differences in cardiovascular parameters between T2D patients and controls, for South Asians and Europeans, were further examined using ANCOVA with adjustment for age, sex, systolic and diastolic blood pressure and smoking status (currently vs. never or formerly). Ethnic group comparisons in the association of T2D with cardiovascular remodeling were made by adding the interaction term between ethnicity and diabetes status to the model [10]. We assessed the normal distribution of the data visually. All statistical tests were two-sided and *P *< 0.05 was considered significant. Statistical analyses were performed using SPSS 23 (IBM Corp, New York, United States).

## Results

### Clinical characteristics and adiposity parameters

In the present study, 48 of the 50 European T2D patients who participated in the trial NCT01761318 [17] were included (n = 1 was excluded because of type 1 diabetes and n = 1 was excluded because of missing CMR data due to claustrophobia). South Asian T2D patients with age > 65 years who participated in the trial NCT02660047 [18] were excluded in this study, as we were unable to enroll South Asian healthy controls in this age category due to the high prevalence of cardiometabolic risk factors among older South Asian individuals. In the current study, 33 of the 47 South Asian T2D patients were included (n = 6 were excluded because of age > 65 years, subsequently n = 6 were excluded due to significant coronary artery disease and n = 2 due to mitral valve insufficiency and/or stenosis on CMR).

In total, the present study comprised 131 individuals (n = 81 patients with T2D and n = 50 healthy controls). In the European cohort (n = 77), 48 patients [mean ± SD age: 59.5 ± 6.6 years, diabetes duration: 10.7 ± 6.2 years, 28 (58%) men] and 29 controls [mean ± SD age: 57.6 ± 7.8 years, 15 (52%) men] were included. The South Asian study population (n = 54) consisted of 33 patients [51.3 ± 9.0 years, diabetes duration: 15.1 ± 9.4 years, 12 (36%) men] and 21 controls [48.3 ± 8.1 years, 6 (29%) men]. Baseline characteristics are presented in Table 1. Whereas the T2D and control groups were similar according to age and sex distribution for both the South Asian and European cohort, the South Asian compared with the European study population was younger (50.1 ± 8.7 vs. 58.8 ± 7.0 years, *P *< 0.001) and consisted of more women [18/54 (33%) vs. 43/77 (56%) men, *P *= 0.013]. The European and South Asian control groups were similar regarding smoking status (*P *= 0.297), systolic and diastolic blood pressure (*P *= 0.467 and 0.973, respectively), triglycerides (*P *= 0.582), total cholesterol (*P *= 0.285), LDL-cholesterol (*P *= 0.848) and HbA1c (*P *= 0.925), except that high-density lipoprotein (HDL)-cholesterol was lower among the South Asians (*P *= 0.010). For the South Asian as compared with the European T2D patients, diabetes duration was longer (*P *= 0.012), the daily insulin dose tended to be higher (*P *= 0.056), the urinary albumin/creatinine ratio was higher (*P *= 0.045) and total cholesterol was lower (*P *= 0.046), whereas HbA1c was similar (*P *= 0.801).

**Table 1:** Clinical characteristics and adiposity parameters

	Europeans		South Asians	
	T2D (n = 48)	Controls (n = 29)	T2D (n = 33)	Controls (n = 21)
Age, years	59.5 ± 6.6	57.6 ± 7.8	51.3 ± 9.0	48.3 ± 8.1
Men, no. (%)	28 (58%)	15 (52%)	12 (36%)	6 (29%)
Current smoker, no. (%)	9 (19%)	1 (3%)	5 (15%)	3 (14%)
BSA, m^2^	2.1 ± 0.2*	1.9 ± 0.2	1.9 ± 0.2*	1.7 ± 0.2
BMI, kg/m^2^	32 ± 4*	24 ± 3	30 ± 4*	24 ± 3
Waist circumference, cm	110 ± 9*	87 ± 9	101 ± 10*	82 ± 7
Men	109 ± 8	91 ± 7	103 ± 8	86 ± 8
Women	112 ± 10	82 ± 9	100 ± 11	81 ± 7
Waist-hip ratio	1.03 ± 0.07*	0.88 ± 0.08	0.96 ± 0.09*	0.86 ± 0.07
Men	1.05 ± 0.06	0.93 ± 0.04	1.00 ± 0.08	0.93 ± 0.05
Women	1.00 ± 0.07	0.83 ± 0.07	0.94 ± 0.08	0.84 ± 0.07
Systolic blood pressure, mmHg	141 ± 15*	126 ± 12	141 ± 21*	124 ± 14
Diastolic blood pressure, mmHg	87 ± 9*	80 ± 8	85 ± 11	80 ± 12
Heart rate, beats/min	71 ± 12*	59 ± 9	69 ± 12*	61 ± 8
Triglycerides, mmol/L	2.2 ± 1.3*	1.0 ± 0.4	1.9 ± 1.5*	0.9 ± 0.3
Total cholesterol, mmol/L	4.8 ± 1.0*	5.7 ± 1.1	4.4 ± 1.0*	5.4 ± 0.8
HDL-cholesterol, mmol/L	1.3 ± 0.3*	1.9 ± 0.5	1.2 ± 0.3*	1.6 ± 0.3
LDL-cholesterol, mmol/L	2.6 ± 0.9*	3.3 ± 1.0	2.2 ± 0.9*	3.4 ± 0.7
Glycated hemoglobin, mmol/mol	65.5 ± 10.8*	35.5 ± 2.7	66.2 ± 11.3*	35.5 ± 2.4
Serum creatinine, μmol/L	70 ± 18	76 ± 14	67 ± 17	73 ± 18
Urinary albumin/creatinine ratio, mg/mmol	0.7 (0.0; 2.7)	–	1.9 (0.5; 6.4)	–
Microalbuminuria, no. (%)	9 (19%)	–	10 (30%)	–
Macroalbuminuria, no. (%)	1 (2%)	–	3 (9%)	–
Diabetes duration, years	10.7 ± 6.2	–	15.1 ± 9.4	–
Metformin, no. (%)	48 (100%)	–	32 (97%)	–
Insulin, no. (%)	31 (65%)	–	23 (70%)	–
Insulin dose, units/day	44 (32; 94)	–	78 (45; 108)	–
Lipid lowering drugs, no. (%)	39 (81%)	–	25 (76%)	–
Antihypertensive drugs, no. (%)	37 (77%)	–	20 (61%)	–
ACE-inhibitors, no. (%)	17 (35%)	–	8 (24%)	–
Total body fat, %	36.7 ± 9.3*	26.9 ± 7.3	37.3 ± 9.1*	32.3 ± 7.1
Men	29.7 ± 3.6	21.9 ± 3.1	27.3 ± 4.9	23.3 ± 4.2
Women	46.4 ± 5.0	32.2 ± 6.7	42.5 ± 5.7	36.0 ± 4.0
Abdominal SAT, cm^2^	346 ± 125*	200 ± 69	344 ± 128*	248 ± 109
Men	277 ± 93	173 ± 55	309 ± 109	204 ± 109
Women	442 ± 97	228 ± 73	364 ± 136	265 ± 107
VAT, cm^2^	207 ± 75*	76 ± 34	152 ± 48*	73 ± 30
Men	214 ± 63	89 ± 31	158 ± 49	94 ± 19
Women	197 ± 89	62 ± 32	148 ± 48	65 ± 29

Mean ± SD, medians (interquartile range) or numbers (percentages) are presented. *P < 0.05 vs. controls. Microalbuminuria and macroalbuminuria: urinary albumin/creatinine ratio (ACR) 3–30 mg/mmol and > 30 mg/mmol, respectively. Missing values: n = 1 for total body fat in the South Asian T2D group

*ACE* angiotensin-converting enzyme, *BMI* body mass index, *BSA* body surface area, *HDL and LDL* high-density and low-density lipoprotein, *VAT and SAT* visceral and subcutaneous adipose tissue

In both the European and South Asian populations, obesity and adipose tissue parameters, blood pressure, cholesterol and glycemic levels were higher in the T2D compared with the control group (Table 1). Differences in obesity and adipose tissue parameters between the T2D and control group in the European and South Asian population were 7.8 kg/m^2^ (6.1; 9.5) and 6.2 kg/m^2^ (4.1; 8.3) for BMI, 24 cm (20; 28) and 19 cm (14; 24) for waist circumference, 0.15 (0.11; 0.18) and 0.10 (0.05; 0.14) for waist-hip ratio, 9.8% (5.8; 13.8) and 5.0% (0.3; 9.7) for total body fat percentage, 146 cm^2^ (96; 197) and 96 cm^2^ (28; 164) for abdominal SAT and 131 cm^2^ (102; 160) and 79 cm^2^ (55; 102) for VAT, respectively.

### Association between T2D and cardiovascular parameters in Europeans and South Asians

**Fig. 1:**
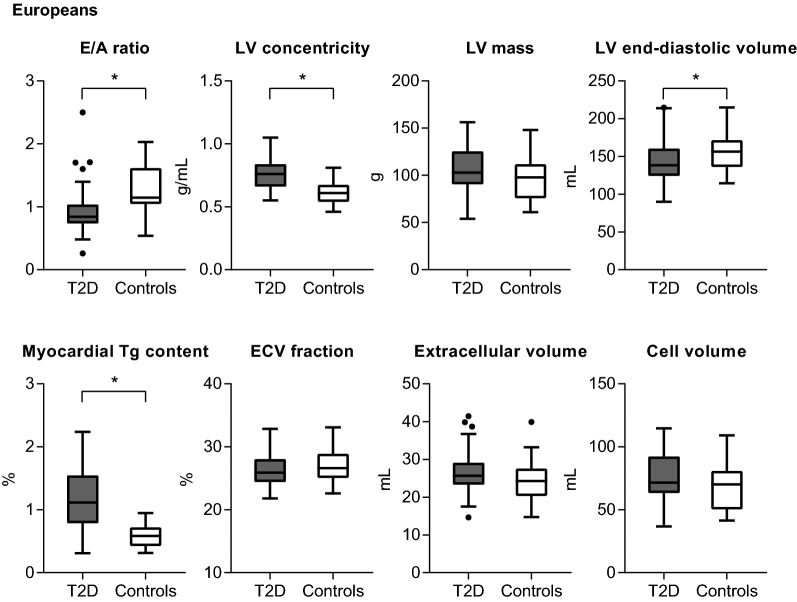
Cardiovascular parameters (boxplots depicting median, interquartile ranges and outliers) in European T2D patients (n = 48) and controls (n = 29) (*P < 0.05). In European T2D patients, the E/A ratio was lower than in the controls, LV concentricity was higher in parallel with a lower LV end-diastolic volume, and the myocardial triglyceride content was higher. *E/A* ratio of the transmitral early and late peak filling rate, *ECV* extracellular volume, *LV* left ventricular, *Tg* triglyceride

**Fig. 2:**
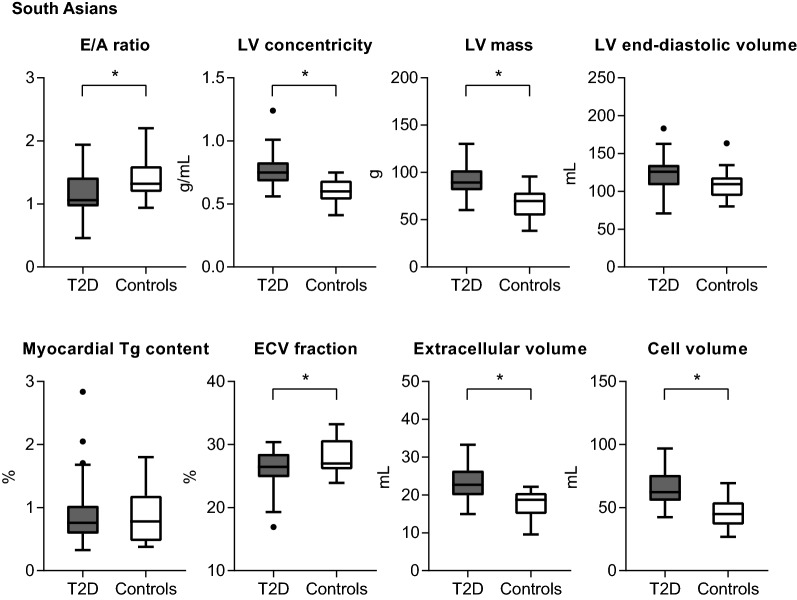
Cardiovascular parameters (boxplots depicting median, interquartile ranges and outliers) in South Asian T2D patients (n = 33) compared with controls (n = 21) (*P < 0.05). In South Asian T2D patients, the E/A ratio was lower than in the controls and LV concentricity was higher in parallel with a higher LV mass. As both the LV extracellular volume and myocardial cell volume were higher, the ECV fraction was slightly lower in South Asian T2D patients than in controls. Abbreviations as in Fig. 1

**Fig. 3:**
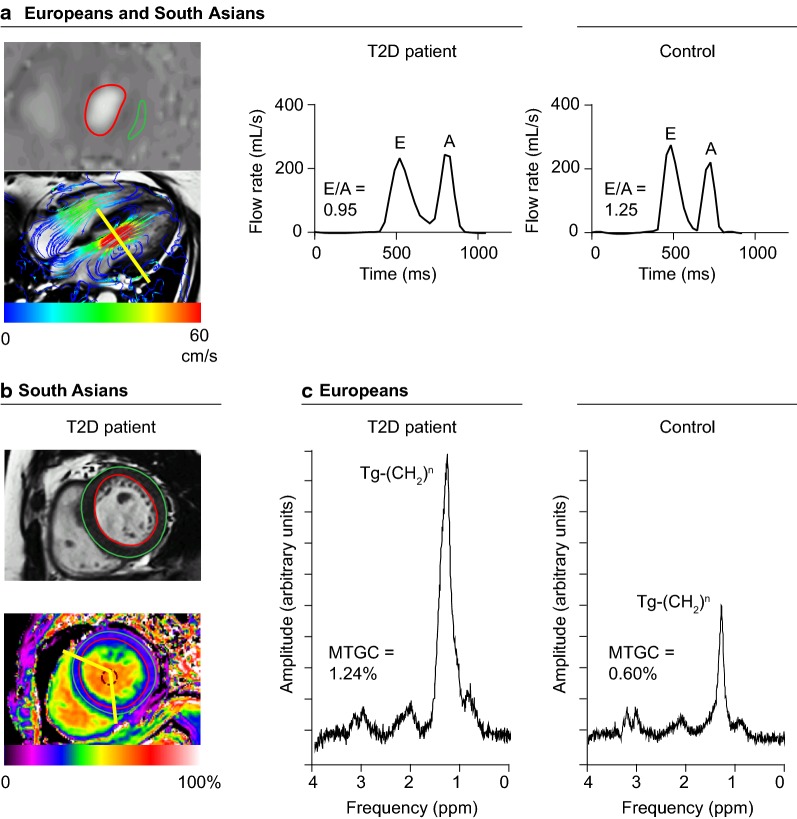
Diabetic cardiomyopathy phenotype in Europeans and South Asians. **a** Impaired LV diastolic function, as indicated by a lower ratio of the transmitral early and late peak filling rate (E/A) was identified as a common characteristic of diabetic cardiomyopathy in Europeans and South Asians. The E/A ratio was measured using 4D velocity-encoded MR after retrospective mitral valve tracking and through-plane motion correction (left image). An example of the transmitral flow rate curve in a T2D patient (South Asian 62-year-old woman with E/A: 0.95) and control (South Asian 57-year-old women with E/A: 1.25) is provided (right images). **b** In South Asian but not in European T2D patients the LV mass, measured on short-axis cine (upper image), was higher than in the control group and the extracellular volume fraction, measured in the mid-ventricular septum using T1 mapping (lower image), was decreased. In the presented South Asian T2D patient, LV mass was 97 g and extracellular volume fraction was 27%. **c** Among Europeans but not among South Asians the myocardial triglyceride content was different for T2D patients compared with controls. An example of cardiac proton-magnetic resonance spectroscopy (^1^H-MRS) in a 45-year-old woman with T2D (left image) and in a 48-year-old non-diabetic woman (right image) of European descent is provided (myocardial triglyceride content (MTGC): 1.24% and 0.60%, respectively)

In univariable analyses, for both Europeans and South Asians, T2D patients as compared with controls had LV structural alterations (higher LV concentricity), with impairments in LV diastolic function (lower E/A ratio and higher LV filling pressure) and aortic stiffness (higher aortic pulse wave velocity) (Tables 2 and 3). The E dec peak, Ea, LV compliance and GLS were impaired in the T2D patients compared with the controls among Europeans but not among South Asians. Cardiovascular parameters for the T2D patients and controls are presented in Figs. 1, 2 and 3.

**Table 2:** Cardiovascular parameters in Europeans

	T2D (n = 48)	Controls (n = 29)
LV diastolic function		
E, mL/s	326 ± 97	352 ± 55
A, mL/s	366 ± 71*	301 ± 75
E/A ratio	0.93 ± 0.38*	1.24 ± 0.36
E dec peak, mL/s^2^ ×10^−3^	− 2.7 ± 1.0*	− 3.2 ± 0.7
Ea, cm/s	5.9 ± 1.7*	7.7 ± 1.9
Estimate of LV filling pressure, mmHg	7.6 ± 2.6*	5.1 ± 1.7
Estimate of LV compliance, mL/mmHg	21.3 ± 9.4*	33.4 ± 11.2
GLSR-E, 1/s	0.78 ± 0.23	0.87 ± 0.28
GCSR-E, 1/s	1.15 ± 0.37	1.22 ± 0.37
LV systolic function		
Ejection fraction, %	55 ± 5	56 ± 6
GLS, %	− 19.3 ± 2.7*	− 21.1 ± 3.3
GCS, %	− 26.1 ± 4.5	− 26.5 ± 3.9
Hemodynamics		
Stroke volume, mL	78 ± 17*	88 ± 17
Cardiac output, L/min	5.4 ± 1.0	5.2 ± 1.1
Cardiac index, L/min/m^2^	2.5 ± 0.4*	2.7 ± 0.5
Aortic stiffness		
Aortic pulse wave velocity, m/s	8.5 ± 2.2*	7.5 ± 1.5
LV structure		
End-diastolic volume, mL	142 ± 29*	156 ± 26
Mass, g	107 ± 23	96 ± 24
Concentricity, g/mL	0.76 ± 0.12*	0.61 ± 0.09
Myocardial tissue characteristics		
Myocardial Tg content, %	1.19 ± 0.53*	0.58 ± 0.18
Native T1, ms	1197 ± 44*	1230 ± 28
Extracellular volume fraction, %	26.3 ± 2.5	26.9 ± 2.7
Extracellular volume, mL	27 ± 6	24 ± 5
Cell volume, mL	75 ± 18	67 ± 18

Mean ± SD. *P < 0.05 vs. controls. Missing values in the T2D group: n = 1 for all flow-derived LV diastolic function parameters, n = 3 E dec peak, n = 1 GLSR-E, n = 1 aortic pulse wave velocity, n = 1 myocardial Tg content, n = 4 native T1, n = 5 extracellular volume, cell volume, fibrosis volume; in the control group: n = 1 for all flow-derived LV diastolic function parameters

*Ea* early peak diastolic mitral septal tissue velocity, *E and A* transmitral early and late peak filling rate, *E dec peak* peak deceleration slope of E, *GLS and GLSR-E* global longitudinal strain and early peak diastolic strain rate, *GCS and GCSR-E* global circumferential strain and early peak diastolic strain rate, *LV* left ventricular, *Tg* triglyceride

**Table 3:** Cardiovascular parameters in South Asians

	T2D (n = 33)	Controls (n = 21)
LV diastolic function		
E, mL/s	339 ± 109	333 ± 67
A, mL/s	302 ± 64*	242 ± 54
E/A ratio	1.15 ± 0.37*	1.42 ± 0.36
E dec peak, mL/s^2^ ×10^−3^	− 2.9 ± 1.2	− 3.2 ± 0.7
Ea, cm/s	5.9 ± 2.0	6.8 ± 1.9
Estimate of LV filling pressure, mmHg	6.9 ± 3.0*	5.0 ± 1.6
Estimate of LV compliance, mL/mmHg	20.5 ± 9.0	24.0 ± 7.7
GLSR-E, 1/s	0.88 ± 0.22	0.98 ± 0.16
GCSR-E, 1/s	1.32 ± 0.34	1.31 ± 0.24
LV systolic function		
Ejection fraction, %	58 ± 6	60 ± 5
GLS, %	− 20.4 ± 2.8	− 21.7 ± 1.7
GCS, %	− 27.6 ± 4.3	− 27.6 ± 3.4
Hemodynamics		
Stroke volume, mL	70 ± 14	66 ± 12
Cardiac output, L/min	4.8 ± 1.0*	4.0 ± 0.7
Cardiac index, L/min/m^2^	2.5 ± 0.4	2.3 ± 0.3
Aortic stiffness		
Aortic pulse wave velocity, m/s	7.9 ± 2.0*	6.5 ± 1.1
LV structure		
End-diastolic volume, mL	123 ± 25	110 ± 19
Mass, g	93 ± 20*	66 ± 15
Concentricity, g/mL	0.77 ± 0.14*	0.60 ± 0.10
Myocardial tissue characteristics		
Myocardial Tg content, %	0.93 ± 0.54	0.84 ± 0.43
Native T1, ms	1255 ± 37	1263 ± 42
Extracellular volume fraction, %	26.2 ± 3.0*	28.2 ± 2.6
Extracellular volume, mL	23 ± 5*	18 ± 4
Cell volume, mL	66 ± 16*	45 ± 11

Mean ± SD. * *P *< 0.05 vs. controls. Missing values in the T2D group: n = 1 native T1 and extracellular volume, cell volume, fibrosis volume; in the control group: n = 1 for myocardial Tg content. For abbreviations see Table 2

In multivariable analyses, in both European and South Asian T2D patients the E/A ratio remained lower and LV concentricity persisted to be higher compared with controls, but there were no differences in the E dec peak, GLS or aortic pulse wave velocity (Table 4). Furthermore, in multivariable analyses, the LV filling pressure was increased or tended to be higher in European and South Asian T2D patients, respectively, compared with their controls. In European but not in South Asian T2D patients, additionally, Ea and LV compliance were lower in comparison with their controls (*P *= 0.095 and 0.008 for interaction by ethnicity, respectively). In European T2D patients the stroke volume was reduced, with a slight decrease in cardiac index, whereas in South Asian T2D patients cardiac index was preserved in comparison with their control groups (*P *= 0.012 for interaction by ethnicity). In European T2D patients the LV concentricity was increased in parallel with lower end-diastolic volumes (*P *= 0.001 for interaction by ethnicity), whereas in South Asian T2D patients the increased LV concentricity was in parallel with a higher LV mass (*P *= 0.005 for interaction by ethnicity). In South Asian but not in European T2D patients compared with their controls, the myocardial cell and extracellular volumes were higher (*P *= 0.003 and 0.071 for interaction by ethnicity, respectively). Because of a larger increase in the myocardial cell volume than in the extracellular volume, the extracellular volume fraction was slightly lower in the South Asian T2D patients than in the controls (*P *= 0.114 for interaction by ethnicity). In European but not South Asian T2D patients, the myocardial triglyceride content was higher and native T1 was lower in comparison with their controls (*P *= 0.002 and 0.078 for interaction by ethnicity, respectively).

**Table 4:** Association between T2D and cardiovascular parameters

	Adjusted mean difference (95% CI) between T2D patients and controls		*P* value for interaction by ethnicity
	Europeans (n = 77)	South Asians (n = 54)	
LV diastolic function			
E/A	− 0.20 (− 0.36, − 0.04)	− 0.20 (− 0.36, − 0.03)	0.480
E dec peak, mL/s^2^ ×10^−3^	0.35 (− 0.13, 0.83)	− 0.03 (− 0.54, 0.49)	0.576
Ea, cm/s	− 1.1 (− 1.9, − 0.4)	− 0.4 (− 1.4, 0.6)	0.095
LV filling pressure, mmHg	2.3 (1.0, 3.6)	1.0 (− 0.4, 2.4)	0.300
LV compliance, mL/mmHg	− 12.9 (− 18.6, − 7.2)	− 1.5 (− 6.1, 3.1)	0.008
LV systolic function			
GLS, %	0.6 (− 0.9, 2.1)	1.3 (− 0.2, 2.8)	0.708
Hemodynamics			
Stroke volume, mL	− 10 (− 19, − 1)	3 (− 4, 11)	0.007
Cardiac output, L/min	0.3 (− 0.3, 0.8)	0.8 (0.3, 1.3)	0.067
Cardiac index, L/min/m^2^	− 0.2 (− 0.4, 0.0)	0.1 (− 0.1, 0.3)	0.012
Aortic stiffness			
Aortic pulse wave velocity, m/s	0.2 (− 0.8, 1.2)	0.6 (− 0.2, 1.4)	0.761
LV structure			
End-diastolic volume, mL	− 20 (− 34, − 7)	11 (0, 22)	0.001
Mass, g	− 1 (− 11, 8)	22 (15, 30)	0.005
Concentricity, g/mL	0.10 (0.04, 0.15)	0.14 (0.07, 0.21)	0.574
Myocardial tissue characteristics			
Myocardial Tg content, %	0.59 (0.35, 0.84)	0.10 (− 0.20, 0.41)	0.002
Native T1, ms	− 25 (− 47, − 3)	− 6 (− 31, 18)	0.078
Extracellular volume fraction, %	1.0 (− 0.3, 2.2)	− 1.9 (− 3.4, − 0.4)	0.114
Extracellular volume, mL	− 1 (− 2, 4)	4 (2, 7)	0.071
Cell volume, mL	− 3 (− 10, 4)	17 (12, 23)	0.003

Adjusted for age, sex, ethnicity, smoking status (currently vs. never or previously), systolic and diastolic blood pressure. *P* value for the interaction by ethnicity in the association between diabetes status and each cardiovascular parameter. For details on missing values see Table 2 and 3. For abbreviations see Table 2

## Discussion

In both Europeans and South Asians, the E/A ratio was lower in relation to T2D, whereas global systolic and diastolic strain parameters and aortic pulse wave velocity were similar in the T2D patients and controls in multivariable analysis. Furthermore, South Asian T2D patients had a higher LV mass with a lower extracellular volume fraction, whilst European T2D patients had a higher myocardial triglyceride content than their control group.

### LV functional and structural remodeling in T2D

In our study, in both the European and South Asian population, T2D was associated with reduced LV diastolic function and increased LV concentricity. LV diastolic function in T2D may be impaired due to disturbances in myocardial substrate utilization and myocardial energetics [11, 27]. More recently, the pro-inflammatory state in T2D has been proposed as an important contributing factor to the increased diastolic LV stiffness [28]. LV diastolic function may be most susceptible to energy shortage [29], whereas abnormalities in LV systolic function may develop once myocardial energetics are substantially disturbed. Furthermore, in our study, T2D was not associated with an increase in aortic stiffness, although oxidative stress due to hyperglycemia and vascular inflammation have been related to impairments in aortic function [30, 31]. Possibly, aortic stiffening, similar as LV systolic dysfunction, may arise in more advanced T2D.

The prevalence of diastolic dysfunction in asymptomatic T2D patients has been reported to be at least 50% [32]. Notably, several studies have indicated that GLS, in addition to diastolic function parameters, may be impaired in T2D patients [14, 33–35]. Also, abnormalities in GLS have been demonstrated even in T2D patients with normal diastolic function and, in this regard, some argue that reduced LV longitudinal contractility rather than diastolic dysfunction should be regarded as the first marker of diabetic cardiomyopathy [36]. Nonetheless, in multivariable analysis, we did not observe a reduction in GLS in T2D patients as compared with controls, neither in the European nor in the South Asian population.

Importantly, the increased LV concentricity in European T2D patients was due to a reduction in LV end-diastolic volume, whereas the higher LV concentricity among South Asian T2D patients was related to an increase in LV mass. These observations imply that the higher LV concentricity in T2D may be the result of impaired LV diastolic function (with therefore incomplete LV relaxation, reduced LV filling and lower LV end-diastolic volumes) and/or LV hypertrophic remodeling [37]. In the Multi-Ethnic Study of Atherosclerosis (MESA) study, the relationships of T2D and impaired fasting glucose to LV mass, end-diastolic volume and stroke volume differed between white, black, Hispanic and Chinese individuals [38]. Similar to our study, the MESA study reported a lower LV end-diastolic volume in white T2D patients, whereas an increased LV mass in relation to T2D, after adjustment for demographic and anthropomorphic factors, was solely observed in the black and Hispanic groups [38]. Notably, it has been suggested that LV hypertrophy in T2D may be partly due to excessive sympathetic activity, driven by insulin resistance [39]. In this regard, we speculate that LV mass was increased in South Asian but not in European T2D patients, in part because of the slightly higher degree of insulin resistance. Furthermore, population studies have shown that South Asian individuals are susceptible of developing T2D at younger age than other ethnic groups [40]. Accordingly, in our study, the diabetes duration was longer for the South Asian compared with the European population. The difference in diabetes duration may have contributed to the increased LV mass as observed in the South Asian but not in the European T2D patients.

A previous community-based study reported that T2D may be more detrimental for cardiac function among South Asians compared with other ethnic groups [10]. Nevertheless, in our study, the degree of LV diastolic dysfunction seemed to be slightly higher for the European than for the South Asian T2D patients, as indicated by the lower Ea and LV compliance and significantly higher LV filling pressure in relation to T2D in the European but not in the South Asian cohort. Obesity as well as T2D has been shown to be associated with LV diastolic dysfunction [41, 42]. The relatively high degree of adiposity in the European T2D patients in comparison with their controls may have accounted for the slightly higher degree of LV diastolic dysfunction than in the South Asian T2D patients.

### Role of myocardial steatosis in diabetic cardiomyopathy

Both in imaging studies in T2D patients and in histological studies in diabetic mice, T2D has been demonstrated to be associated with myocardial steatosis [13, 43–45]. Myocardial lipid overstorage is presumed to induce LV diastolic impairments due to the lipotoxic effects of intermediates such as diacylglycerol and ceramide [44–46]. Also, ectopic fat accumulation in the myocardium may reflect altered substrate utilization due to insulin resistance, with an increase in fatty acid oxidation [11, 27]. In keeping with previous findings, we observed a twofold increase in myocardial triglyceride content in T2D patients as compared with healthy controls within the European cohort [13]. Interestingly, the myocardial triglyceride content seemed to be increased in the healthy controls of South Asian descent compared with those of European origin, to similar levels as in the T2D patients, despite the strict screening for both T2D and prediabetes. Myocardial triglyceride content in South Asians as compared with Europeans has been previously assessed in young men and middle-aged overweight men, where no differences were found despite the observed higher systemic insulin resistance in those studies [47, 48]. Presumably, myocardial and systemic insulin sensitivity may not be necessarily interrelated. Accordingly, in a previous study on the relation between myocardial triglyceride content and LV diastolic function in healthy controls, increasing age, but not whole-body insulin resistance, correlated with myocardial steatosis and reduced LV diastolic function [49]. Also, it has to be noted that myocardial triglyceride content in non-diabetic individuals reflects the rate of fatty acid uptake as well as oxidation, and may therefore be dependent on many other factors in addition to myocardial insulin sensitivity, for example the amount of circulating fatty acids, dietary carbohydrate intake, plasma glucose availability and muscular glycogen stores [50]. Although our data reaffirm the potential role of myocardial steatosis in diabetic cardiomyopathy, our results suggest that myocardial triglyceride accumulation may be present in non-diabetic individuals as well as T2D patients.

### Role of diffuse fibrosis in diabetic cardiomyopathy

Previously, myocardial diffuse fibrosis as indicated by an increased extracellular volume fraction has been demonstrated in obese adolescents with or without T2D, independent of blood pressure [16]. Interestingly, in a recent population-based study, the extracellular volume fraction was found to be reduced in T2D patients [51]. It has been suggested that antihypertensive medication, especially angiotensin-converting enzyme (ACE) inhibitors, may regress myocardial fibrotic remodeling in response to hypertension [52]. Our South Asian but not European T2D study population had an increased LV mass, paralleled by a reduced extracellular volume fraction. A majority of the patients were prescribed antihypertensive drugs, with almost halve of them using ACE-inhibitors. This may explain the considerable increase in myocardial cell volume in response to increased LV afterload, with only a slight increase of the LV extracellular volume. Although myocardial diffuse fibrosis may contribute to LV functional impairments in the early stages of T2D, our results suggest that the extracellular volume fraction may not be necessarily increased in patients with longstanding T2D with concomitant antihypertensive medication use.

### Limitations

Due to the cross-sectional design of our study, we cannot address issues of causality in the association of T2D with cardiovascular parameters. Our work should be considered a hypothesis-generating study and larger prospective studies are required to confirm the differential impact of T2D on LV structure and myocardial tissue characteristics between Europeans and South Asians. Due to the relatively low sample size, we could not perform subgroup analyses, for example by sex. It has been recognized that T2D in women may be more detrimental for cardiac function as compared with men [53]. The South Asian as compared with the European study population was younger and, importantly, consisted of more women. Possibly, this may have introduced bias. Furthermore, there is a well-established relation between insulin resistance and high blood pressure [54, 55], which impedes the recruitment of T2D patients without hypertension. In this regard, to assess the relation of T2D to cardiovascular remodeling separately from hypertension, we adjusted for systolic and diastolic blood pressure; nonetheless, we cannot exclude residual confounding. Ideally, diabetic cardiomyopathy is studied in the absolute absence of coronary artery disease. However, a cardiac stress test was not part of our screening procedure. Furthermore, findings of previous studies indicate that several coexisting factors affect myocardial remodeling in individuals with T2D. For example, it has been demonstrated that cardiac dysfunction is worse in obese T2D patients than in individuals with T2D but without overweight [56]. Also, nutrition may interfere with the process of myocardial inflammation and fibrosis in T2D [57]. Therefore, differences in body weight and potential differences in diet between the T2D patients and controls may have partly confounded the observed relation of T2D to myocardial remodeling.

## Conclusions

LV diastolic dysfunction was identified as a common characteristic of diabetic cardiomyopathy among South Asians and Europeans. Importantly, increased LV mass was solely observed among South Asian T2D patients, whereas differences in myocardial triglyceride content between T2D patients and controls were only present in the European cohort. This study comprised detailed phenotyping of cardiovascular remodeling in T2D patients, in both South Asians and Europeans. Our results suggest that the diabetic cardiomyopathy phenotype may differ between subsets of T2D patients, for example across ethnicities. Therefore, further research on tailored strategies for T2D management may be warranted.

## Data Availability

The datasets generated during and/or analyzed during the current study are available from the corresponding author on reasonable request.

## Abbreviations

ACE: angiotensin-converting enzyme
BMI: body mass index
BSA: body surface area
CMR: cardiovascular magnetic resonance
E/A: ratio of transmitral early and late peak filling rate
Ea: early peak diastolic mitral septal tissue velocity
E dec peak: peak deceleration slope of the transmitral early peak filling rate
ECG: electrocardiography
ECV: extracellular volume
eGFR: estimated glomerular filtration rate
FA: flip angle
FOV: field of view
GCS: global circumferential strain
GCSR-E: global circumferential early peak diastolic strain rate
GLS: global longitudinal strain
GLSR-E: global longitudinal early peak diastolic strain rate
^1^H-MRS: proton-magnetic resonance spectroscopy
HDL and LDL: high-density and low-density lipoprotein
LV: left ventricle/ventricular
MOLLI: modified Look-Locker inversion recovery
MTGC: myocardial triglyceride content
SAT: subcutaneous adipose tissue
SENSE: sensitivity encoding
T2D: type 2 diabetes
TE: echo time
Tg: triglyceride
TR: repetition time
VAT: visceral adipose tissue
venc: velocity encoding
